# Combination of autophagy inducer rapamycin and oncolytic adenovirus improves antitumor effect in cancer cells

**DOI:** 10.1186/1743-422X-10-293

**Published:** 2013-09-23

**Authors:** Pei-Hsin Cheng, Serena Lian, Robin Zhao, Xiao-Mei Rao, Kelly M McMasters, Heshan Sam Zhou

**Affiliations:** 1 Department of Pharmacology and Toxicology, University of Louisville School of Medicine, Louisville, KY 40292, USA; 2 Department of Surgery, University of Louisville School of Medicine, Louisville, KY 40292, USA; 3 James Graham Brown Cancer Center, University of Louisville School of Medicine, Louisville, KY 40292, USA; 4 Department of Microbiology and Immunology, University of Louisville School of Medicine, Louisville, KY 40292, USA

**Keywords:** Oncolytic adenovirus, Replication, Cyclin E, Autophagy, Rapamycin

## Abstract

**Background:**

Combination of oncolytic adenoviruses (Ads) and chemotherapy drugs has shown promising therapeutic results and is considered as a potential approach for cancer therapy. We previously have shown that autophagy may generate decomposed cellular molecules that can be used as nutrition to support virus replication in cancer cells. In this study, we evaluated a unique combination of the novel oncolytic Ad-cycE with rapamycin, an autophagy inducer and first-line chemotherapeutic drug.

**Methods:**

The combination of oncolytic Ad-cycE and the autophagy inducer rapamycin was assessed for enhanced antitumor effect. We also evaluated the combined effects of rapamycin and Ad-cycE on cancer cell viability. The interaction between Ad-cycE and rapamycin was analyzed with Calcusyn (Biosoft, Ferguson, MO).

**Results:**

We show that rapamycin induces autophagy, enhances Ad E1A expression and increases Ad oncolytic replication. Combination of rapamycin and Ad-cycE elicits stronger cytotoxicity than single treatment alone. The analyzed data indicates that the Ad-cycE and rapamycin combination has a significantly synergistic antitumor effect.

**Conclusions:**

Our study provides a new insight into vector development and demonstrates the novel roles of autophagy in adenovirus replication. The combination of autophagy-induced chemotherapy and oncolytic virotherapy may be a new approach to improve future cancer treatment.

## Background

Oncolytic virotherapy with *E1b55K*-deleted adenoviruses (Ads) has been applied to human clinical trials in the United States and approved for the commercial use in China [1-5]. The selective oncolytic effects can be achieved by a small quantity of viruses that spread to surrounding tumor cells, therefore contributing to an interesting drug platform [6,7]. Considering the viral tropism, respiratory oncolytic Ads should have a high potential for lung cancer therapy [8]. However, lung cancer is generally difficult to treat with oncolytic viruses, and there are few recorded successful trials due to the cancer’s propensity to metastasize and the irregular shape of most tumors. Therefore, developing combination strategies to target human lung cancer with improved oncolytic Ads would allow for more effective treatment.

In clinical treatments, oncolytic Ads are generally used with the first-line chemotherapy drugs, and the combination treatments have exhibited high therapeutic efficiency and improved safety [9]. However, the interaction mechanism between chemotherapy drugs and viruses has not been well characterized. Selecting drugs for combination therapies based on the understanding of the interaction between Ads and drugs definitely will benefit the feasibility of this strategy. In our previous study, we have shown that the treatment of the autophagy inducer rapamycin increased the Ad yields and the autophagy inhibitor 3-methyladenine (3-MA) reduced Ad replication [10]. Our studies have also shown that autophagy may generate decomposed cellular molecules as nutrition to support Ad replication. Thus, an autophagy inducer may improve virus oncolytic therapy.

Autophagy is a process involving the lysosomal degradation and recycling of cellular proteins and cytoplasmic organelles [11]. Environment stressors such as nutrient starvation and pathogen infection induce autophagy. Autophagy initiates from membrane structures called phagophores [11-13], which engulf cellular and cytoplasmic components, followed by elongation and recruitment of microtubule-associated protein 1 light chanin 3 (LC3) to form the characteristic double-membrane autophagosome. Cytoplasmic form LC3-I and lipidated form LC3-II are two forms of LC3 post-transcriptionally produced in cells [14-16]. LC3 is immediately processed into LC3-I after synthesis. During the autophagy process, LC3-I is cleaved by cysteine protease Atg4 to generate lipidated form LC3-II that localizes on autophagosome membranes [13,17]. The amount of LC3-II or the LC3-II/LC3-I ratio can be used to estimate the degree of autophagosome formation [14,15,18]. Autophagosomes eventually fuse into lysosomes to form autolysosomes, in which the inner components undergo the degradation process and produce amino acids and fatty acids for reuse in cells. Rapamycin, the inhibitor of the mammalian target of rapamycin (mTOR) [19,20], has been shown to induce autophagy and inhibit proliferation of malignant glioma cells [21]. Autophagy is negatively regulated by the PI3K-AKT-mTOR pathway. Via inhibiting the negative regulation of mTOR signaling, rapamycin indirectly enhances autophagy.

Using a tumor-specific promoter to regulate Ad E1A expression is a general effort to control vector selective replication in cancer cells and cause oncolysis. The proteins encoded by the *E1a* region, expressed immediately after infection, then modulate the cell cycle, recruit cellular proteins, and produce viral proteins to process viral DNA replication [22]. However, all known tumor-specific promoters are relative weak compared with the native promoter of the Ad *E1a* gene [23,24]. In addition, Ad infection can cause strong repression of most cellular promoters, as indicated in our published microarray study [25]. Vectors driven by tumor-specific promoters generally elicit low potency and do not work as efficiently as *dl*1520, which contains the native *E1a* promoter and is applied in current tumor treatments [23,24]. However, the native *E1a* promoter does not exhibit selectivity and therefore has side effects, such as virus replication in noncancerous cells [26,27]. Obviously, the selection of promoters in vector construction should consider the negative effects imposed by virus infection on those promoters. We thus have constructed a novel *E1b*-deleted oncolytic Ad-cycE, in which Ad *E1a* gene is driven by the cyclin E promoter. Cyclin E is known to regulate DNA replication and promote the S-phase entry [28,29]. Cyclin E overexpression is frequently detected in many types of cancers, including lung cancer [30]. Recent studies also showed that overexpression of cyclin E can trigger lung cancers in transgenic mice [31,32]. Our previous studies revealed that the replication of *E1b55K*-deleted Ads is significantly repressed in G_0_-arrested normal cells [33,34], in which the cyclin E promoter is restricted. We have also demonstrated that the activity of cyclin E promoter in cancer cells is further augmented after Ad infection [33,35]. As the replication of *E1b55K*-deleted Ad-cycE depends on the activation of cyclin E promoter, Ad-cycE replication may be enhanced in cancer cells and repressed in normal cells.

In this study, we applied novel tumor-specific Ad-cycE and rapamycin in combination to enhance oncolytic effects. We show that Ad-cycE is competent to replicate in human lung cancer cells but not in the normal lung cells and that the combination of oncolytic Ad-cycE and the autophagy inducer rapamycin elicits synergistic inhibition effects. We also reveal that rapamycin increases Ad E1A expression and virus production. Our studies have clearly shown that autophagy inducers as chemotherapeutic agents are capable of increasing adenoviral replication and oncolysis. Thus the combination of autophagy-associated chemotherapy and oncolytic virotherapy may be a new approach to improve future cancer treatment.

## Methods

### Cell lines and culture conditions

HEK 293 (ATCC no. CRL-1573), WI-38 human lung fibroblast (ATCC no. CCL-75), MCF10A human mammary epithelial (ATCC no. CRL-10317), MDA-MB-231 human breast cancer (ATCC no. HTB-26), A549 (ATCC no. CCL-185) and H1299 (ATCC no. CRL-5803) human lung cancer cell lines were purchased from the American Type Culture Collection (Rockville, MD). WI-38 human lung fibroblast cell line has the properties of primary cells with a finite lifetime of 50 population doublings [36]. MCF10A human mammary epithelial cell line is an immortalized but non-transformed human breast epithelial cell line [37,38]. WI-38 cells were cultured in minimal essential medium (MEM) Alpha GlutaMAX with 0.1 mM non-essential amino acids and 1.0 mM sodium pyruvate. MCF10A cells were cultured in DMEM/F12K with 20 ng/ml EGF, 0.5 μg/ml Hydrocortisone, and 10 μg/ml insulin. HEK 293, A549 and MDA-MB-231 cells were cultured in DMEM. All media were supplemented with 5% (for MCF10A cells) or 10% (for the other cells) fetal bovine serum (FBS) and penicillin/streptomycin (100 U/ml). Cells were cultured in a 5% CO_2_ incubator at 37°C. All cell culture reagents were obtained from Gibco BRL (Bethesda, MD).

### Adenoviral vectors

**Figure 1:**
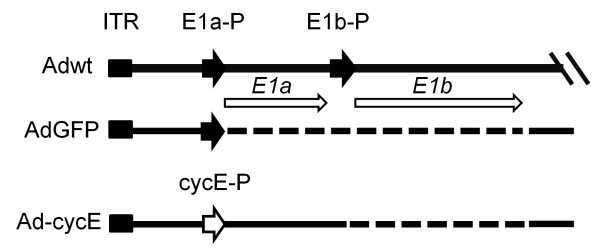
**Structure of the Ads.** The wild-type Ad (Adwt) with the *E1a* and *E1b* genes and their endogenous promoters is shown at the top. The left inverted terminal repeat (ITR), the promoters for *E1a* gene and *E1b* genes (E1a-P and E1b-P) and the *E1a* and *E1b* open reading frames are indicated. The solid lines represent Adwt regions in these viruses, and the dashed lines represent the deleted regions. AdGFP contains the complete deletion of *E1a* and *E1b* regions and their promoters. Ad-cycE contains the deletion of *E1b* region and a cyclin E promoter (cycE-P) was inserted to replace the deleted *E1a* promoter.

Figure 1 depicts the structures of the adenoviruses applied in this study. Wild-type adenovirus type 5 (Adwt, ATCC no. VR-5) was used as a replication-competent control. AdCMV/GFP, an Ad vector with *E1* deletion carrying a green fluorescent protein (GFP), was used as a replication-defective control [39]. Ad-cycE is a novel *E1b*-deleted oncolytic vector carrying a human cyclin E promoter driving an intact E1A expression cassette. The endogenous *E1a* promoter was deleted and a human cyclin E promoter (GenBank ID: X95406 [40]) was inserted to replace the deleted *E1a* promoter in Ad-cycE. Therefore, Ad-cycE contains a human cyclin E promoter to control *E1a* open reading frames (ORF). The details of Ad-cycE construction will be reported separately in our preparing report. All of the vectors created and used in this study are based on the backbone of wild-type Ad type 5.

### Cytotoxicity assay

Cells were seeded into 24-well plates at a density of 2.5 × 10^4^ (cells/well) and cultured under the indicated conditions. After 72 hours, Cytotoxicity was assessed with crystal violet staining [41]. Cells were fixed and stained with 1% crystal violet followed by washing with water to remove excess dye. The dye was solubilized with 2% SDS and the absorbance of the solubilized stain was measured at 590 nm using a Synergy HT Multi-Mode Microplate Reader (Bio-Tek, Winooski, VT). The OD values were quantitated into the cell viability % by the formula, cell viability % = (OD value of experimental group / OD value of control group) × 100%. Rapamycin and viruses were diluted with corresponding culture media. The 0 nM control group was treated with the diluents and was calculated as 100% of cell viability in the assay [42].

### Analyses of combination effects of rapamycin and Ad-cycE

In this study, an additive effect refers to a combined effect of drugs that produces the sum of their individual effects; synergism is the combined effect of drugs which is greater than the sum of individual effects, and antagonism is the combined effect of drugs which is less than the sum of individual effects [43,44]. The combined effects of rapamycin and Ad-cycE on cell viability were analyzed with the median-effect methods of Chou and Talalay [45] using CalcuSyn software (Biosoft, Ferguson, MO). The combination index (CI) values were used to evaluate the interaction between the drug and virus. For the fraction of virus affected combination index (Fa-CI) plot analysis, a CI < 1 is defined as synergism, a CI = 1 is defined as an additive effect, and a CI > 1 is defined as antagonism. The data were confirmed with the isobologram method [46,47]. The diagonal curves connecting the x- and y-axes were calculated from single treatments to represent the additive effect for the theoretical combinations of two treatments at the specific effective doses. If the data points fall on the lower left of the diagonal, the combination is regarded as synergism. If the experimental data points of the drug combination fall on the diagonal, the combination is regarded as an additive effect. If the data points fall on the upper right of the diagonal, the combination is regarded as antagonism.

### Viral titration

Cells were seeded into 6-well plates at a density of 2 × 10^5^ (cells/well) and treated under the indicated conditions. Total infected cells and culture supernatants were collected at 48 h postinfection (p.i.) and lysed to release virus particles with three cycles of freezing and thawing. The viral yields were determined by the infective unit method as described previously [48,49]. HEK 293 cells were seeded in 96-well plates at a density of 10^3^ (cells/well) and then infected with 10-fold serially diluted viruses. CPE was recorded and scored after incubation for 7 days.

### Western blot analysis

Cells were harvested and lysed with CDK2 lysis buffer (20 mM Tris pH 7.5, 150 mM NaCl, 5 mM MgCl_2_, 0.5% Nonidet P-40, 0.1% Brij 35, 5 mM sodium glycerophosphate, 1 mM sodium vanadate, 1 mM dithiothreitol). The Western blot analyses were performed as described previously [34]. 25 μg of cell lysates were electrophoresed through 10 or 12% SDS-polyacrylamide gels and transferred onto an Immobilon-P Membrane (Millipore, Billerica, MA). The primary antibodies used in this study were rabbit anti-LC3 and actin (Sigma, St. Louis, MO), mouse anti-adenovirus type 5 E1A (BD Pharmingen, San Jose, CA), and rabbit anti-adenovirus type 5 antibody (Abcam, Cambridge, MA). Actin was used as an internal control. The membranes were then incubated with anti-mouse immunoglobulin G (IgG) or anti-rabbit IgG peroxidase-linked species-specific whole antibody (GE Healthcare, Piscataway, NJ). Chemiluminescent detection was performed with ECL reagents according to the supplier’s recommendations (GE Healthcare). The scanned band intensity was quantitated by Gel-pro Analyzer 4.0 software (Media Cybernetics, Bethesda, MD) according to the manufacturer’s tutorial. Densitometric value for each band was expressed as integrated optical density (I.O.D.) and normalized with actin. The results were reported as the ratios of normalized band intensities of LC3-II to LC3-I.

### Statistical analyses

All above experiments, except specifically indicated, were repeated at least three times. Quantitation results were reported as means ± standard deviation (S.D.). The Pearson correlation coefficient (*r*) was used to evaluate the correlations between the rapamycin concentrations and cell viability percentages by SAS software, Version 9.3 (SAS Institute Inc., Cary, NC) [50,51]. Statistical difference of the combination experiment was assessed with a Student's *t*-test. Statistical significance of difference was set at p < 0.05.

## Results

### Selective replication of Ad-cycE in cancer cells

Figure 1 depicts the structures of the adenoviruses applied in this study. Adwt was used as a replication-competent control. AdCMV/GFP, a vector with *E1a* and *E1b* deletion carrying a green fluorescent protein (GFP), was used as a replication-defective control. Our previous reports indicated that the cyclin E promoter is more active in lung cancer cells than in normal lung cells and oncolytic *E1b*-deleted Ad infection further elevates the promoter activation [33,35]. Thus, we replaced the native *E1a* promoter with the cyclin E promoter to generate Ad-cycE, a novel *E1b*-deleted oncolytic vector.

**Figure 2:**
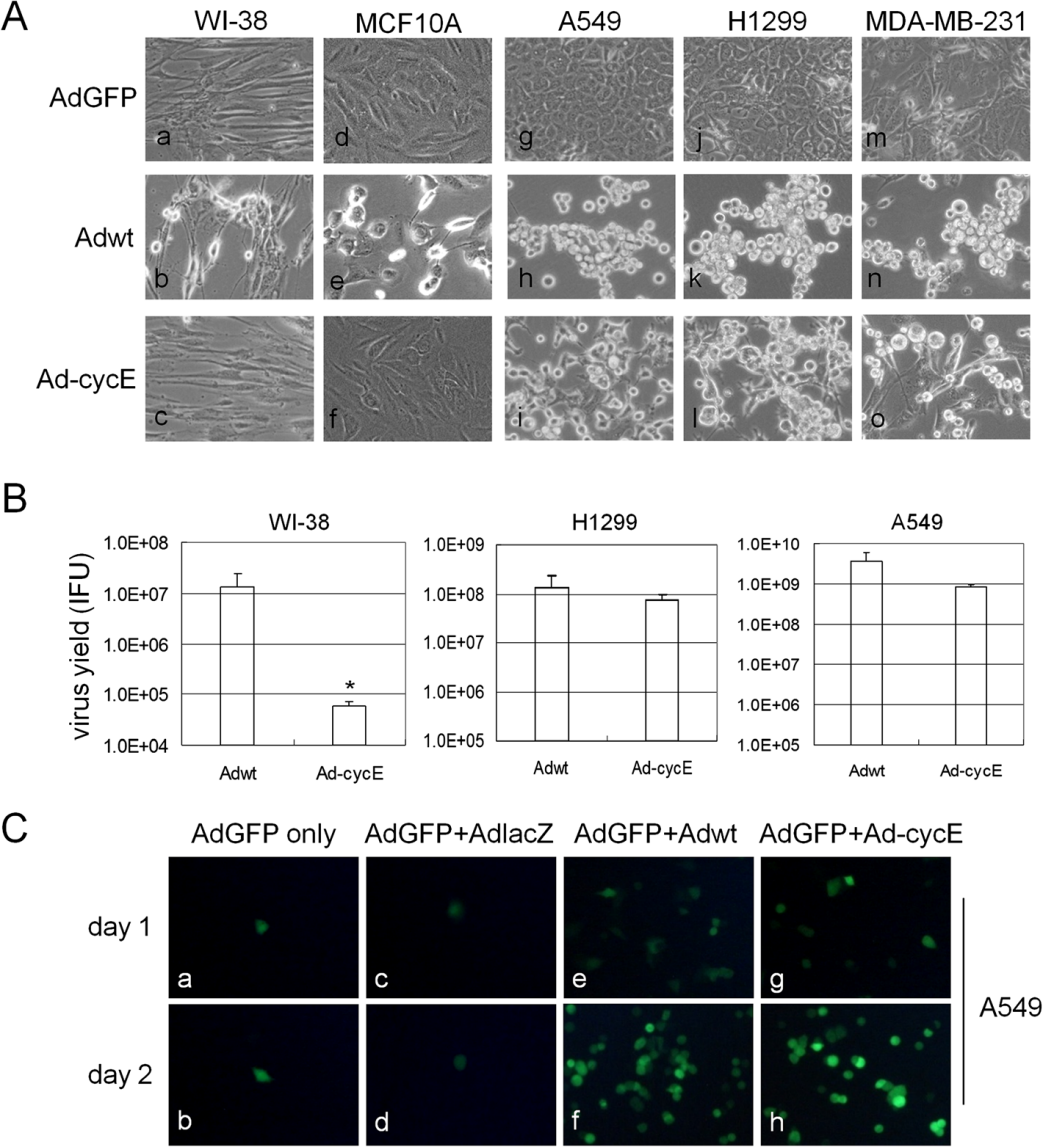
**Selective oncolytic replication of Ad-cycE. (A)** WI-38, MCF10A, A549, H1299 and MDA-MB-231 cells were infected with AdGFP, Adwt, or Ad-cycE at 5 MOI. CPE was observed at 48 hr or 72 hr p.i and photographed with an inverted microscope Olympus CKX41. **(B)** Viral yields produced in WI-38, H1299 and A549 cells were determined at 72 hr p.i. with the infection unit method. The values represent the means ± S.D. of independent triplicate. * P<0.05, Student’s *t*-test. **(C)** A549 cells were co-infected with 5 MOI AdGFP and mock-infection, AdlacZ, Adwt or Ad-cycE. All fluorescent microscopy is taken at day 1 and day 2 p.i. with an Olympus IX50 microscope (original magnification of ×100).

To determine the selectivity of Ad-cycE for cancer cells, we first examined the cytotoxicity of Ad-cycE on normal and cancer cell lines. WI-38 human lung fibroblast, MCF10A mammary epithelial, A549 and H1299 lung cancer and MDA-MB-231 breast cancer cells were infected with AdGFP, Adwt or Ad-cycE at 5 MOI. Ad-cycE replicated in A549 and H1299 human lung cancer, and MDA-MB-231 breast cancer cells and caused cytopathic effect (CPE) similar to that of Adwt (Figure 2A, comparing panel h and i, k and l, n and o). The CPE became visible at 48 hr and 72 hr p.i., showing that the infected cells became circular and detached from the cell monolayer. Unlike Adwt, which indistinguishably induced CPE in noncancerous WI-38 human lung fibroblast and MCF10A mammary epithelial cells, Ad-cycE selectively induced CPE in all tested cancer cells (Figure 2A, comparing panel b and c, e and f).

WI-38, A549 and H1299 cells were infected with Adwt or Ad-cycE at 5 MOI and the total infected cells and culture supernatants were collected at 72 hr to examine the production of infectious virus particles. Figure 2B revealed that in noncancerous WI-38 human lung fibroblast cells, the virus yield of Adwt was significantly higher than that of Ad-cycE (P = 0.04); in H1299 human lung cancer cells, there was no significant difference between the virus yield of Adwt and Ad-cycE (P = 0.2); in A549 human lung cancer cells, Adwt (3.7 × 10^9^) and Ad-cycE (8.3 × 10^8^) achieved a high level of virus yield, indicating the replication property of Ad-cycE in lung cancer cells. The aggregate data from this experiment shows that Ad-cycE can selectively replicated in and efficiently destroy cancer cells but poorly replicates in noncancerous cells.

Previous studies indicate that replication-competent oncolytic Ads could produce the essential Ad E1 proteins to support the replication of replication-defective *E1*-deleted Ads cotransduced *in vitro* or *in vivo *[35,52,53]. To further verify selective replication capability of Ad-cycE in cancer cells, A549 human lung cancer cell line with constitutive cyclin E production [33] was chosen for the following experiment. A549 cells were infected with 5MOI AdGFP alone, or AdGFP plus an additional Ad (AdGFP+AdlacZ, AdGFP+Adwt or AdGFP+Ad-cycE). Figure 2C showed that the non-replicative AdGFP maintained the original level of infectivity at day 1 and day 2 (comparing panel a and b). Also, with coinfection of AdGFP and non-replicative AdlacZ, the fluorescent cell numbers did not change (comparing panel c and d). Yet with the addition of Adwt (comparing panel e and f) or Ad-cycE (comparing panel g and h), we detected an increase of fluorescent cell numbers from day 1 to day 2, suggesting that efficacy of Ad-cycE replication in cancer cells is comparable with the wild-type Ad.

### Rapamycin induces autophagy and inhibits lung cancer cell growth

**Figure 3:**
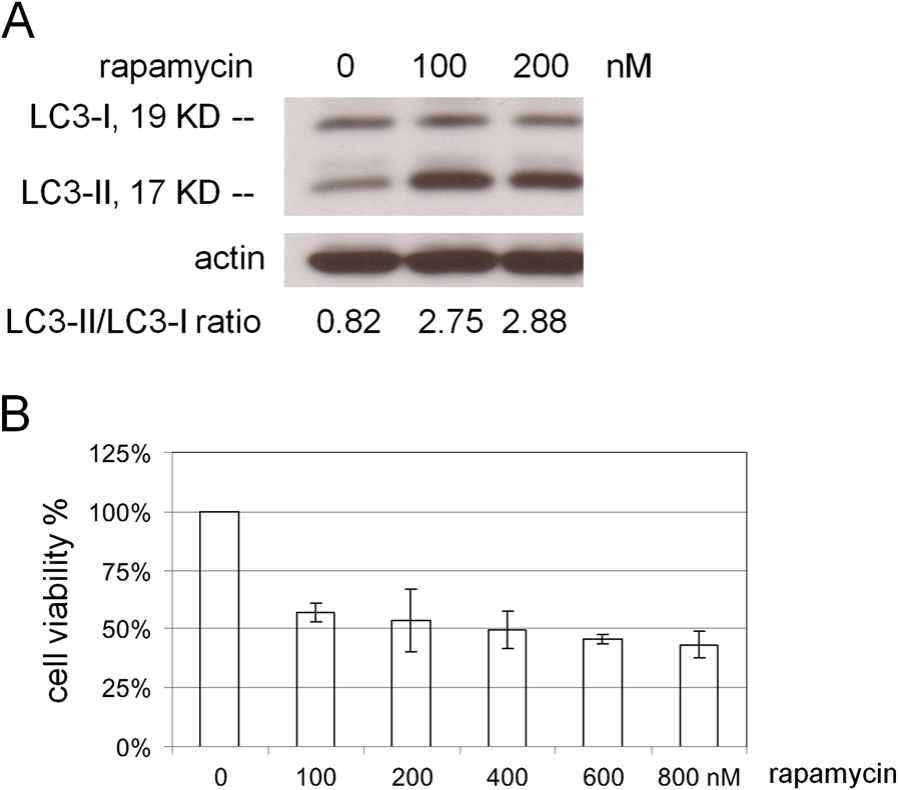
**Effects of rapamycin on cytotoxicity and autophagy.** A549 cells were treated with 0 nM, 100 nM, and 200 nM ramamycin (LC Laboratories, Woburn, MA) and collected at 24 hr after treatment. **(A)** Cell lysates were immunoblotted for LC3 and actin. Actin was used as a loading control. The values indicate the ratios of normalized band intensities of LC3-II to LC3-I. **(B)** A549 cells were treated with 0 nM, 100 nM, 200 nM, 400 nM, 600 nM and 800 nM ramamycin. The cell viability % was determined at 72 hr after treatment. The values of cell viability % represent the means ± S.D. of independent quadruplicate compared with the 0 nM-control group.

Rapamycin has been shown to induce autophagy and inhibit proliferation of malignant glioma cells [21]. We investigated whether rapamycin can induce autophagy in A549 lung cancer cells. The cells were treated with 0 nM, 100 nM, and 200 nM rapamycin for 24 hr. Western blot was used to determine the conversion of LC3-I to LC3-II, which is one of the representative characteristics of autophagy activation. LC3 is immediately processed into LC3-I after synthesis. Then the cytoplasmic form LC3-I is cleaved by cysteine protease Atg4 to generate lipidated form LC3-II that specifically localizes to autophagosome membranes [17]. Thus the amount of LC3-II or the LC3-II/LC3-I ratio can be applied to estimate the abundance of autophagosomes [14,15,18]. Figure 3A shows two forms of LC3, the upper band corresponding to LC3-I and lower band corresponding to LC3-II [14]. Compared with the 0 nM-control group (the ratio of LC3-II/LC3-I = 0.82), the 100 nM and 200 nM rapamycin treatments increased the amount of LC3-II and caused the ratio of LC3-II/LC3-I to 2.75 and 2.88, respectively, indicating the induction of autophagy. Next we examined the effect of rapamycin on A549 cell growth. The cells were treated with 0 nM, 100 nM, 200 nM, 400 nM, 600 nM and 800 nM rapamycin for 72 hr. The cell viability was determined with crystal violet staining and quantitated into cell viability percentages. The results showed that rapamycin decreased cell viability in a dose-dependent manner at 72 hr compared to the 0 nM-control group (*r* = −0.69033, P = 0.0002) (Figure 3B).

### Combination of rapamycin and Ad-cycE elicits stronger cytotoxicity than single treatment alone

**Figure 4:**
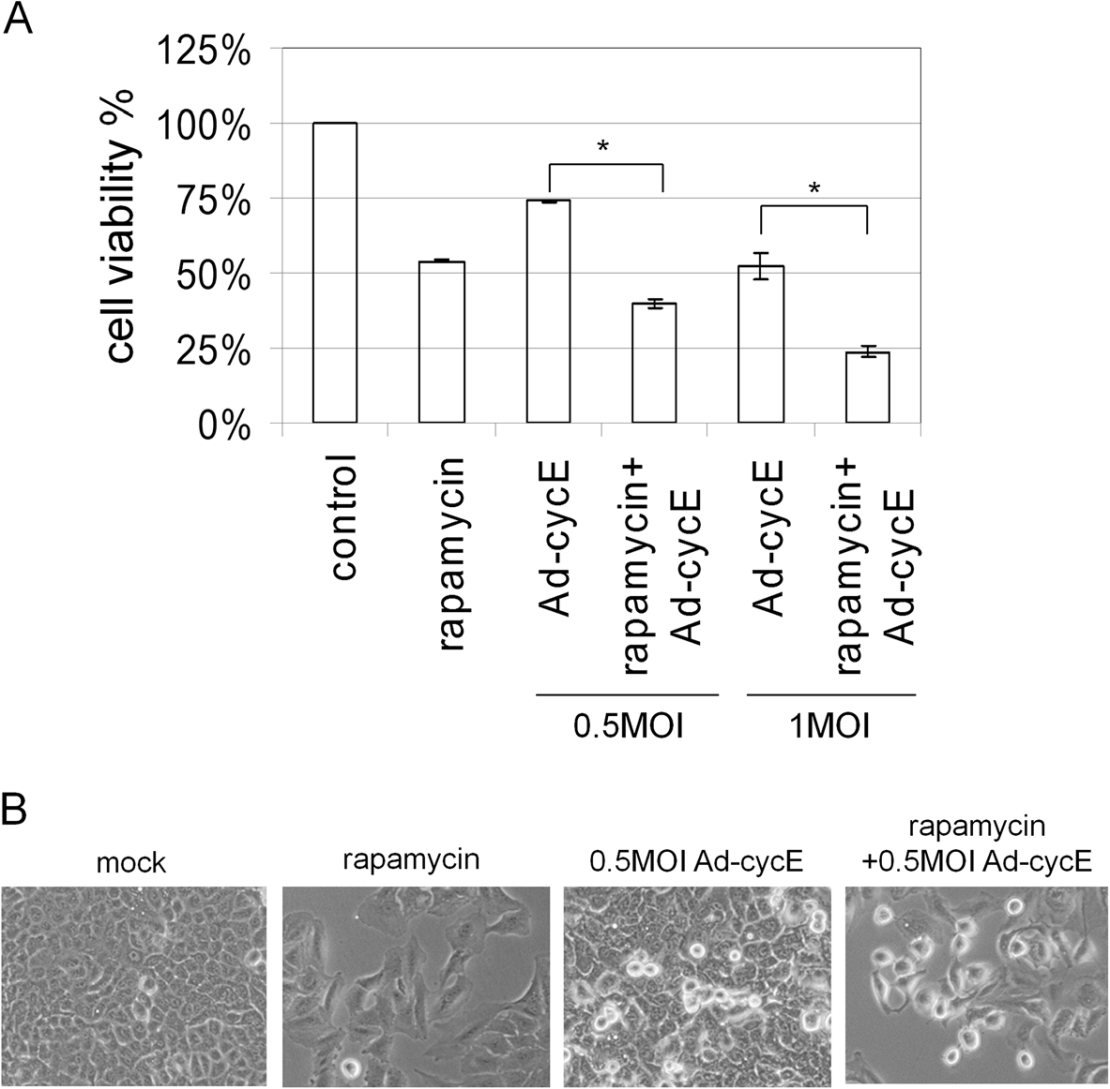
**Effects of combination of rapamycin and Ad-cycE on A549 cells.** A549 cells were non-treated or treated with 200 nM rapamycin alone, 0.5 or 1 MOI Ad-cycE alone, or the combination treatment of 200 nM rapamycin and 0.5 or 1 MOI Ad-cycE. **(A)** The results were quantitated into cell viability %. The values of cell viability % represent the means ± S.D. of independent triplicate compared with the mock-control group. * P<0.05, Student’s *t*-test. **(B)** CPE was photographed at a magnification of x100 at 48 hr p.i.

We first chose 200 nM rapamycin as the working condition and tested the combination effects of rapamycin with different MOIs of Ad-cycE on lung cancer cell growth. Figure 4A shows a difference between the cell viability percentage of treatment with Ad-cycE alone and Ad-cycE in combination with rapamycin. Our results show that Ad-cycE in combination with rapamycin induces greater CPE in A549 lung cancer cells than either treatment alone. The distinction can be clearly seen in both 0.5 MOI Ad-cycE and 1 MOI Ad-cycE. Statistical Student's *t*-tests confirmed the significant difference. Treatments with 200 nM rapamycin or 1 MOI Ad-cycE both resulted in the cell viability of about 50% (53.9% for rapamycin and 52% for Ad-cycE. Combination of Ad-cycE and rapamycin decreased cell viability to 23.6% (P = 0.00000011). We repeated the experiment with even lower dose of Ad-cycE (0.5 MOI), which only is able to induce slight CPE. 0.5MOI Ad-cycE only caused 73.9% of the cell viability (P = 0.0052), but combination with 200 nM rapamycin caused 39.4% of the cell viability (P = 0.0000000002). The cell morphology was photographed with an inverted microscope on day 2 (Figure 4B). These results suggest that rapamycin in combination with Ad-cycE elicits greater cytotoxicity on A549 cells even with a low MOI of Ad-cycE.

### Rapamycin increases Ad E1A expression and oncolytic replication

**Figure 5:**
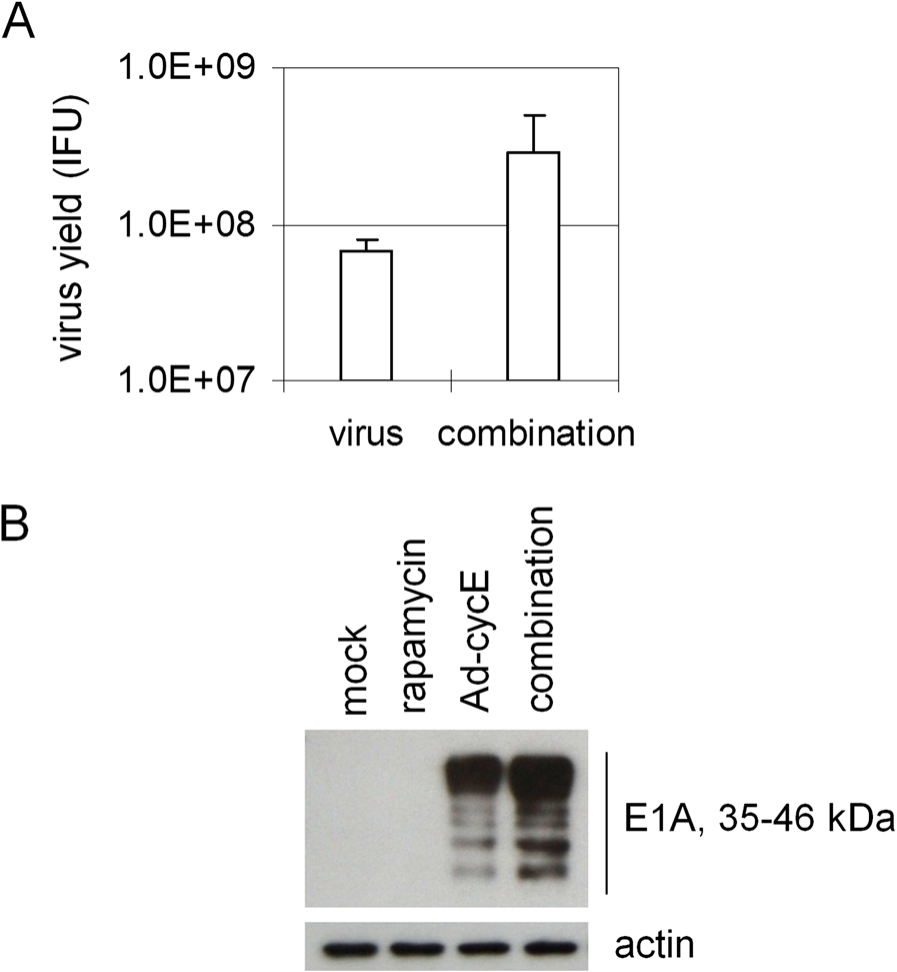
**Effects of rapamycin on the oncolytic replication of Ad-cycE.** A549 cells were non-treated or treated with 200 nM rapamycin alone, 0.5 MOI Ad-cycE alone, combination treatment of 200 nM rapamycin and 0.5 MOI Ad-cycE. **(A)** Viral yields were determined at 48 hr p.i. with the infection unit method. The values represent the means ± S.D. of independent triplicate. **(B)** Cell lysates harvested at 18 hr p.i. were immunoblotted for Ad E1A and actin. Actin was used as a loading control.

The stronger antitumor effect in the combination treatment may be generated from the sum of the effect of two individual treatments or even a synergistic effect (one treatment may increase the efficacy of the other). To understand the mechanism by which rapamycin in combination with Ad-cycE caused stronger antitumor effects, we first examined the production of virus particles, comparing virus alone with the combination groups. Rapamycin treatment led to a 4.25-fold increase in virus yield compared to the virus alone group (Figure 5A). This suggests that rapamycin increases the production of Ad-cycE in A549 cells, resulting in stronger antitumor effects than either drug or virus alone. Next we examined the E1A expression to determine the mechanism by which rapamycin may contribute to the increased production of Ad vectors. E1A is the crucial protein which is expressed immediately after infection and initiates the virus replication cycle [22]. Ad E1A protein expression was examined at 18 hours and identified as multiple bands at 35–46 kDa generated from the alternative splicing of E1A transcripts [54]. As shown in Figure 5B, rapamycin stimulates elevated E1A expression in the combination group when compared to Ad-cycE alone. Taken together, the results suggest that rapamycin increases oncolytic replication of Ad-cycE in A549 cells and enhances E1A expression.

### Combination of rapamycin and Ad-cycE elicits synergistic antitumor effects

**Figure 6:**
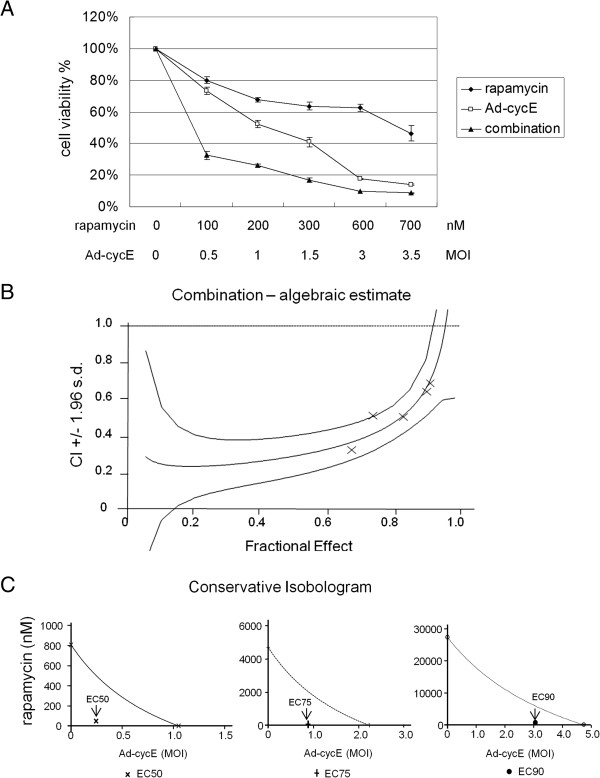
**Analysis by Calcusyn (Biosoft, Ferguson, MO) of the interaction between rapamycin and Ad-cycE on A549 cells.** Cells were treated with rapamycin alone, Ad-cycE alone or combination of both for 96 hr. **(A)** The results were quantitated into cell viability %. The values of cell viability % represent the means ± S.D. of independent triplicate compared with the mock-control group. **(B)** The quantitated cell viability data were analyzed by CalcuSyn software. The X-marks represent the combination index (CI) values of the combination treatment groups. The middle curve line represents the simulated combination index values of the combination treatment groups surrounded by two lines of algebraic estimations of the 95% confidence intervals. **(C)** The effective concentration EC_50_, EC_75_ and EC_90_ refer to the concentration of a drug or combination of drugs that induces 50%, 75% and 90% inhibition of cell viability. In the conservative isobologram plot, the three curves of the expected EC_50_, EC_75_ and EC_90_ additive effect lines for the combination treatments are labeled; the individual points of EC_50_, EC_75_ and EC_90_ for the combination treatments were indicted by arrows and located below their additive interaction lines, respectively.

To determine whether combination of rapamycin and Ad-cycE conducts synergistic antitumor effects, we evaluated the combination treatments with Calcusyn (Biosoft, Ferguson, MO). The combination experiment was performed by adopting the constant ratio drug combination design proposed by Chou and Talalay [45]. A549 human lung cancer cells were treated with rapamycin alone (from 100 nM to 700 nM), Ad-cycE alone (from 0.5 MOI to 3.5 MOI) or a combination of rapamycin (nM) with Ad-cycE (MOI) at the constant ratio of 200:1 for 96 h. Concordant with the results shown in Figure 4, combination of rapamycin and Ad-cycE caused a greater cytotoxicity than either treatment alone (Figure 6A). We then evaluated these quantitated data by fraction affected versus combination index (Fa-Cl) with CalcuSyn software (Biosoft, Ferguson, MO) (Figure 6B). The X-marks represent the combination index (CI) values of the combination treatment groups. The CI values are 0.326, 0.512, 0.506, 0.642, and 0.689 for 100 nM rapamycin plus 0.5 MOI Ad-cycE, 200 nM rapamycin plus 1 MOI Ad-cycE, 300 nM rapamycin plus 1.5 MOI Ad-cycE, 600 nM rapamycin plus 3 MOI Ad-cycE, and 700 nM rapamycin plus 3.5 MOI Ad-cycE, respectively. The middle curve line represents the simulated combination index values of the combination treatment groups surrounded by two lines of algebraic estimations of the 95% confidence intervals. All experimental CI values at the tested ratio were significantly < 1 and between the two confidence lines, indicating synergism of combination treatments.

Since rapamycin and Ad treatments have entirely independent modes of action, the conservative isobologram method [45,55] was also applied here to confirm the above Fa-Cl results. The effective concentration EC_50_, EC_75_ and EC_90_ refer to the concentration of a drug or the combination of the two drugs that induces 50%, 75% and 90% inhibition of cell viability [44]. Figure 6C showed the conservative isobologram plots of EC_50_, EC_75_ and EC_90_, separately. In the conservative isobologram plot, the curve connecting each axis indicates the simulated additive effect for EC_50_, EC_75_ and EC_90_, respectively. The experimental EC_50_, EC_75_ and EC_90_ doses of the combination treatment groups are displayed as the single point indicated by the arrow. The point values of the EC_50_, EC_75_ and EC_90_ for the combination treatments all fall below their diagonal lines for simulated additive effects, indicating that significantly lower doses of rapamycin and Ad-cycE are therapeutically effective when combined. For example, in EC_50_ isobologram, from the simulated curve of the additive effect it shows that to reach 50% inhibition of cell viability requires at least 800 nM rapamycin or 1 MOI Ad-cycE. However, with the combination of rapamycin and Ad-cycE it takes a relatively low dose (50 nM rapamycin plus 0.25 MOI Ad-cycE) to achieve the same efficacy, suggesting combination treatment elicits a greater effect (synergism) than an additive effect. These results in Figure 6A, B and C have all demonstrated that the combination of rapamycin with Ad-cycE elicits a synergistic antitumor effect in A549 human lung cancer cells at the tested concentration ratio.

**Figure 7:**
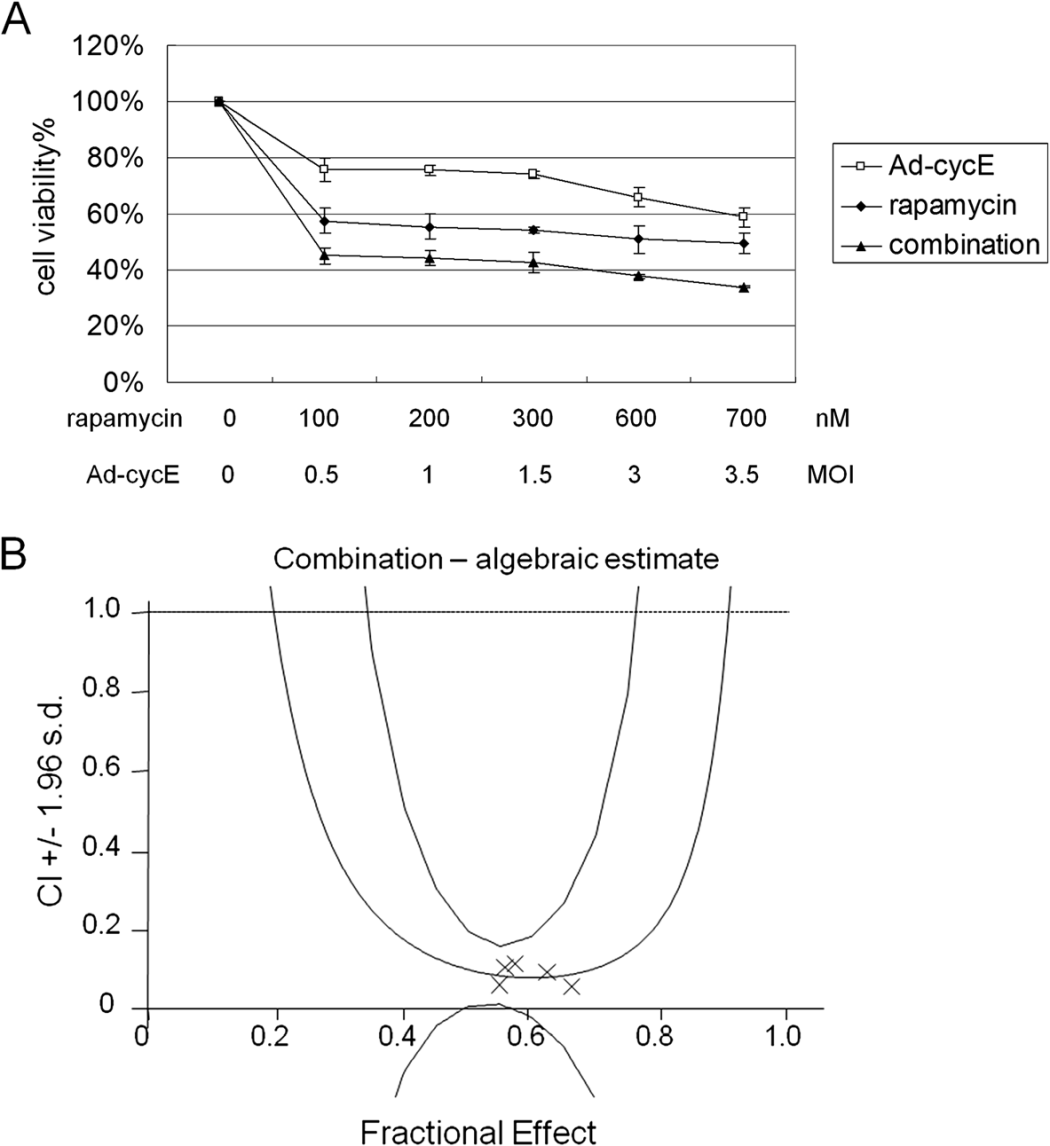
**Effects of combination of rapamycin and Ad-cycE on MDA-MB-231 cells. (A)** Cells were treated with rapamycin alone, Ad-cycE alone or combination of both for 96 hr. The results were quantitated into cell viability %. The values of cell viability % represent the means ± S.D. of independent triplicate compared with the mock-control group. **(B)** The quantitated cell viability data were analyzed by CalcuSyn software. In the fraction affected versus combination index (Fa-CI) plot, all experimental CI values at the tested ratio were significantly < 1 and within the confidence lines.

In addition, we examined the combination effect of rapamycin and Ad-cycE on MDA-MB-231 human breast cancer cell line, which has been reported as a non-permissive cancer cell line for oncolytic Ads replication [33]. As we observed in A549 cells, the combination of rapamycin and Ad-cycE induced a greater cytotoxicity than either treatment alone in MDA-MB-231 cells (Figure 7A) and the therapeutic effect was significantly enhanced by the synergism of combination treatments (Figure 7B).

**Figure 8:**
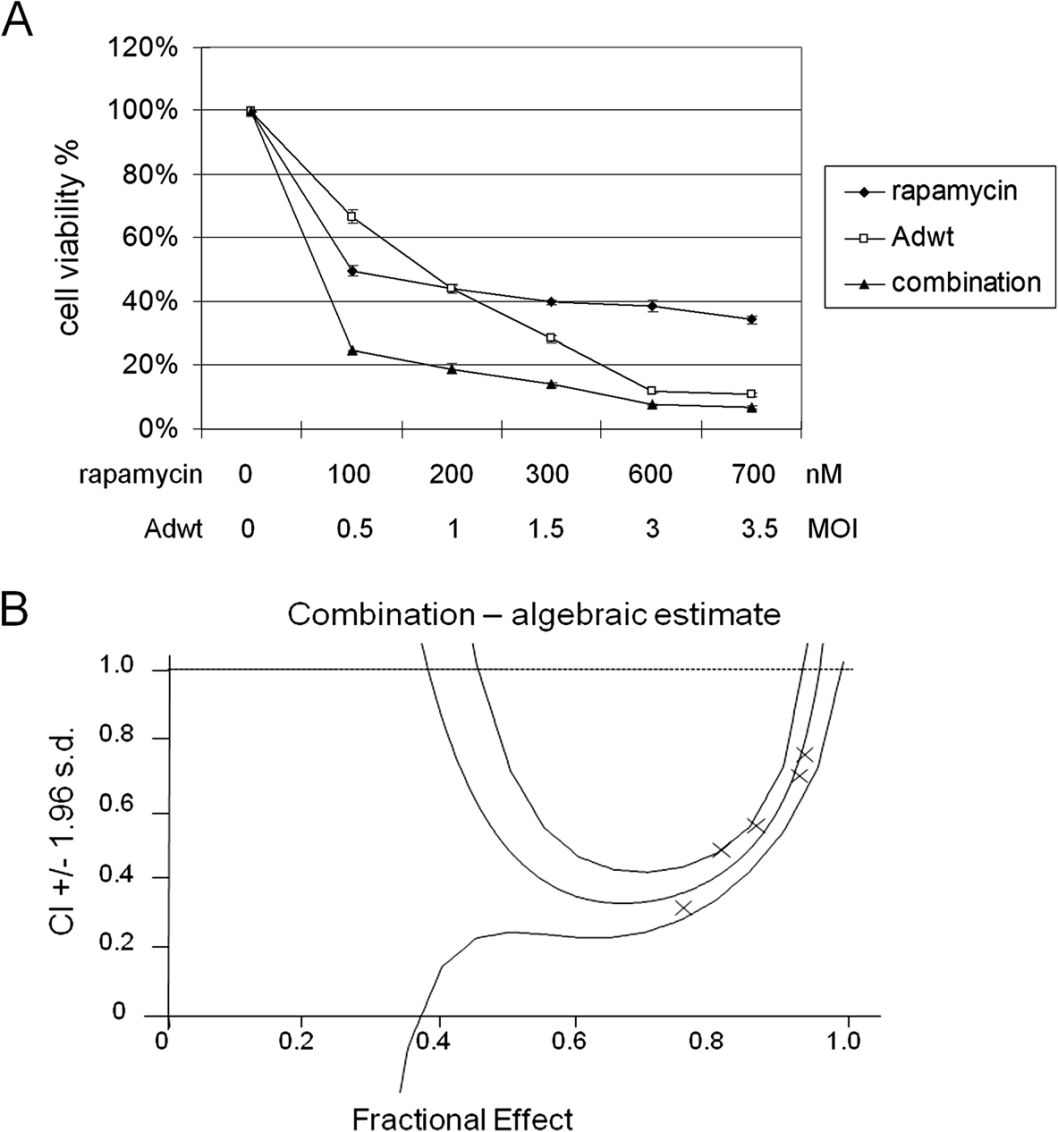
**Effects of combination of rapamycin and wild-type Ad on A549 cells. (A)** Cells were treated with rapamycin alone, Adwt alone or combination of both for 72 hr. The results were quantitated into cell viability %. The values of cell viability % represent the means ± S.D. of independent triplicate compared with the mock-control group. **(B)** The quantitated cell viability data were analyzed by CalcuSyn software. In the fraction affected versus combination index (Fa-CI) plot, all experimental CI values at the tested ratio were significantly < 1 and within the confidence lines.

To determine whether the findings with rapamycin and oncolytic Ad-cycE may apply to wild-type Ad, we tested the same conditions with the combination of rapamycin and Adwt. Consistent to the results shown in Figures 6 and 7, combination of rapamycin and Adwt also caused a greater cytotoxicity than either treatment alone (Figure 8A). Fa-Cl plot showed that all CI values at the tested concentration ratio were significantly < 1 and between the two confidence lines, suggesting the synergism of rapamycin and Adwt (Figure 8B). Our data indicated that the synergism is not only observed in the combination of rapamycin and oncolytic Ad-cycE but also in that of rapamycin and Adwt, suggesting the potential of applying rapamycin to the strategy of combination treatment with the other oncolytic Ads.

## Discussion

Oncolytic virotherapy has shown promising therapeutic results and is considered a potential approach for cancer therapy [56]. The matchless advantage of this approach is that selective oncolytic effects are initiated by a small amount of viruses that spread to the surrounding regions until all cancer cells are destroyed [6]. However, due to the current limitations, virus replication and diffusion are restricted in animal studies and clinical trials when the objectives carry tumors with large masses [49,57,58]. Viruses have difficulty penetrating massive tumors; this may be a reason for disappointing therapeutic outcomes. Developing new strategies to increase virus propagation in tumors is important in improving the efficiency of oncolytic virotherapy.

In our previous study we have shown that autophagy may generate decomposed cellular molecules as nutrients to support virus replication [10]. Therefore we applied the autophagy inducer rapamycin to develop a combination strategy with oncolytic Ad-cycE. First, rapamycin-caused autophagy can generate more nutrients that can be used for building the viral particles [10,59]. Second, autophagy may increase virus particle release from dead cells that may benefit viral spread in tumors [60]. Third, rapamycin has been applied to transplant recipients as an immunosuppressant to prevent organ rejection [61]. The immunosuppressive properties of rapamycin mainly result from the inhibition of leukocyte activity and cytokine expression. Thus, rapamycin as an immunosuppressant may help virus to decrease host antiviral responses and improve virus distribution in tumors. Finally, autophagy-induced cell death has been applied as the new target in chemotherapy [62]. Thus the antitumor effects can be enhanced by both rapamycin-caused autophagy and virus-mediated oncolysis.

We demonstrated that Ad-cycE selectively replicated in cancer cells. Ad-cycE in combination with autophagy-inducer rapamycin further induced synergistic antitumor effects. Rapamycin may also improve oncolytic therapy mediated by other viruses. Studies have shown that an autophagy mechanism is required for hepatitis B virus replication [63], the initiation of hepatitis C virus replication [64] and the promotion of viral replication of the RNA viruses such as poliovirus and rhinovirus [65]. The new role of autophagy to help the virions of adenovirus type 2 (Ad2) to traffic in cells has also been discovered in a recent study [59]. After the virus has been internalized into cells, high level of autophagosomes induced by autophagy are reported to fuse with the early endosomes containing virions and form amphisomes, creating an environment favoring the release of virions into cytosol. Here, we specifically observed that the autophagy inducer rapamycin increased the E1A expression and led to higher Ad-cycE production. In agreement with our finding, Zeng and Carlin (2013) reported that starvation-induced autophagy enhanced the E1A expression and the viral progeny production of Ad2 in human airway epithelial cells [59]. E1A is the crucial protein expressed immediately after infection and regulates the expression of multiple cellular and viral genes to initiate the virus replication cycle [22]. Therefore, we reasoned that autophagy is not only able to generate nutrients for building viral particles, but is also able to increase the E1A expression of Ads, leading to higher virus production and the enhanced combination therapeutic effects.

mTOR pathway has been considered as a determinant regulator in the cellular metabolism [66]. The mTOR inhibitor rapamycin has been reported to elicit diverse and paradoxical effects on the cellular metabolism. Some studies suggested that rapamycin decreases glucose metabolism [67-69] and mitochondrial oxidative functions in mammalian cells [70,71], whereas some others suggested that rapamycin increases glycolysis and oxidative phosphorylation in the targeted cells [72,73]. Fang et al. (2013) pointed out that although detrimental metabolic changes were observed at early stages of rapamycin treatment in mice, the prolonged rapamycin treatment leaded to beneficial metabolic alterations, including increased insulin sensitivity, improved lipid profile and metabolism [74]. Apparently, the discrepancy of those metabolic alternations by rapamycin likely depends on the natures of signaling pathways activated in the cell lines and the duration of treatment [73,74]. Under this circumstance, the relation between the metabolic alterations induced by mTOR inhibition and the adenoviral replication still remains unclear. Some DNA viruses such as adenovirus and human cytomegalovirus stimulate metabolic alternations such as glycolysis in the host cells to generate energy and essential elements for viral replication [75-77]. Besides autophagy, the property of rapamycin to induce metabolic changes may be also utilized by adenovirus to create a beneficial environment for the viral replication.

Based on our previous work with the chemical CDK2 inhibitor roscovitine [39], we noticed that some chemotherapeutic agents with the kinase inhibition properties may inhibit oncolytic Ad replication and thus impair the outcome of oncolytic virotherapy in the combination therapy. It is important to select the chemotherapeutic agents without negative effects on oncolytic viruses when conducting the combination therapy. mTOR regulates several essential signal transduction pathways including the control of cell-cycle progression [66]. As an mTOR inhibitor, one of the key functions of rapamycin is to inhibit cell-cycle progression [78]. Rapamycin is reported to decrease cyclin D1 expression [79], reduce the kinase activity of cyclin D1/CDK4 and cyclin E/CDK2 complexes [80], and block the elimination of the CDK inhibitor p27 [81], leading to cell cycle arrest in G_1_-S-phase [78]. The mechanism(s) by which oncolytic adenoviruses overcome the cell cycle arrest by rapamycin-induced mTOR inhibition requires the further study. Considering the possible negative effects of rapamycin on cell cyclins and cell-cycle progression, autophagy is likely to conduct a very important role for the rapamycin-enhanced virus replication in this study.

## Conclusions

Our studies suggest a novel strategy involving targeting cyclin E overexpression in cancer cells and the properties of autophagy to enhance adenoviral oncolysis that could have a significant impact on clinical outcomes in cancer therapy. The combination of Ad-cycE and rapamycin can be further tested *in vivo* to evaluate the efficacy and efficiency for the clinical setting. Our findings also provide important information for future adenoviral vector development and the combination study for improving oncolytic virotherapy.

## Abbreviations

MOI: Multiplicity of infection; CPE: Cytopathic effect; nM: Nanomolar; DMEM: Dulbecco’s modification of eagle’s medium; FBS: Fetal bovine serum; OD: Optical density; Ad: Adenovirus; Adwt: Wild-type adenovirus.

## Competing interests

The authors declare that they have no competing interests.

## Authors’ contributions

PHC, KMM and HSZ designed the study and drafted the manuscript. PHC, SL, RZ, KMM, and HSZ participated in the revision of the manuscript. PHC, SL, RZ, and XMR carried out the experiments. PHC, KMM, and HSZ participated in the coordination of the study. All authors read and approved the final manuscript.
